# Effects of Adding Transversus Abdominis Plane Block on Intravenous Nalbuphine and Dexmedetomidine for Severely Pre‐Eclamptic Parturients After Cesarean Delivery

**DOI:** 10.1002/kjm2.70027

**Published:** 2025-05-21

**Authors:** Yong Yang, Ya‐Ni Zhang

**Affiliations:** ^1^ Department of Anesthesiology Taizhou Hospital of Zhejiang Province Affiliated to Wenzhou Medical University Taizhou Zhejiang China; ^2^ Department of Operating Room Nursing Taizhou Hospital of Zhejiang Province Affiliated to Wenzhou Medical University Taizhou Zhejiang China

**Keywords:** cesarean section, dexmedetomidine, nalbuphine, severe preeclampsia, transversus abdominis plane block

## Abstract

The use of opioids is frequently associated with the occurrence of adverse effects during cesarean delivery, especially for primiparous women with severe preeclampsia, creating a critical need for investigation of alternative analgesic strategies. The study aims to determine the effects of adding transversus abdominis plane block (TAPB) on patient‐controlled intravenous analgesia (PCIA) of nalbuphine (Nal) and dexmedetomidine (Dex) on severely pre‐eclamptic parturients after cesarean delivery. This is a randomized controlled trial. Severely pre‐eclamptic parturients who were scheduled for elective cesarean delivery with spinal anesthesia were randomly assigned into the TAPB group (TAPB combined with PCIA with Nal and Dex; *n* = 49) and the PCIA group (same block procedures with normal saline followed by PCIA with Nal and Dex; *n* = 51). Results showed that adding TAPB to PCIA with Nal and Dex significantly lowered visual analog scale (VAS) scores at rest at 2 and 6 h, at mobilization at 2, 6, and 12 h after surgery, reduced press time of PCIA, shortened time for first feeding and out‐of‐bed movement, enhanced maternal satisfaction with pain control, and lowered plasma levels of cortisone and norepinephrine. The findings of the study suggest that adding TAPB to PCIA with Nal and Dex could enhance acute recovery with fewer stress responses for severely pre‐eclamptic parturients after cesarean delivery.

## 1 Introduction

Severe pre‐eclampsia is a hypertensive disorder of pregnancy that remains a leading cause of maternal and fetal morbidity and mortality, and spinal anesthesia has been considered the preferred anesthetic option in this high‐risk group of patients [1]. In severely pre‐eclamptic parturients, spinal anesthesia may contribute to an increased risk of hypotension compared to epidural anesthesia, whereas this hypotension is typically easily treated without adding clinically significant differences in outcomes [2]. However, severely pre‐eclamptic parturients are more likely to have a low pain threshold for postcesarean pain resulting from surgical incision and uterine manipulation, thus leading to postpartum depression and delayed secretion of milk [3, 4, 5]. Laboratory findings in pre‐eclamptic women include increased levels of oxidative stress markers, circulating tyrosine kinase 1, and inflammatory activation of leucocytes, indicating that hormonal stress response should be more focused for severely pre‐eclamptic parturients undergoing cesarean section [6]. This creates a critical need for postoperative care, such as complete postcesarean analgesia, hemodynamic stability, and enhanced recovery with less stress response, for severely pre‐eclamptic parturients after cesarean section.

Although long‐acting neuraxial opioids are widely used for postcesarean analgesia largely due to their significant analgesic efficacy and favorable safety profile, their use has been associated with nausea, vomiting, respiratory depression, pruritus, hypothermia, and decreased bowel motility [7]. Multimodal analgesia is widely promoted to enhance recovery and minimize postcesarean opioid use thus tempering the side effects [8]. Transversus abdominis plane block (TAPB), an easy‐to‐operate nerve block receiving acceptance in postoperative analgesia for abdominal surgery, has been studied as part of a multimodal analgesic regimen in a bid to improve postoperative pain management and patient satisfaction, minimizing postoperative opioid use and reducing opioid‐related side effects [9]. Adding TAPB to patient‐controlled intravenous analgesia (PCIA) with sufentanil could provide a good analgesic effect without adding side effects while reducing opioid consumption after cesarean section [10]. In severely pre‐eclamptic parturients, adding TAPB to intrathecal morphine could alleviate postcesarean pain in the first 12 h after surgery and improve maternal satisfaction [11]. Adding TAPB to PCIA with nalbuphine (Nal) has been shown to exhibit a good analgesic effect, thereby reducing the incidence of adverse reactions [12]. Herein, we propose a hypothesis that adding TAPB to PCIA with Nal and dexmedetomidine (Dex) could provide better postcesarean analgesia and enhance acute recovery with less stress response for severely pre‐eclamptic parturients. To prove this hypothesis, we recruited severely pre‐eclamptic parturients who were scheduled for elective cesarean delivery and planned spinal anesthesia to compare postoperative analgesia, acute recovery, stress response, and maternal satisfaction to pain control in two groups arranged to receive adding TAPB or not to PCIA with Nal and Dex.

## 2 Methods

### 2.1 Participant Selection

The study recruited severely pre‐eclamptic parturients who were scheduled for elective cesarean delivery and planned spinal anesthesia at our hospital between February 2023 and February 2024. The inclusion criteria for parturients were as follows: primiparous women, age ranging from 18 to 42 years, gestational age > 28 weeks, severe pre‐eclampsia defined as systolic blood pressure (SBP) of at least 160 mmHg and/or diastolic blood pressure (DBP) of at least 110 mmHg on two occasions, or ≥ 3+ proteinuria with evidence of end organ injury [13], singleton pregnancy, spinal anesthesia, and the Edinburgh Postnatal Depression Scale (EPDS) < 9 scores indicating no depression symptoms [14]. The exclusion criteria were as follows: BMI > 40 kg/m^2^, multiple gestations, history of cesarean section and multiparas, thrombocytopenia, coagulopathy, American Society of Anesthesiologists physical status (ASA) > 4, any contraindication to spinal anesthesia [1], or psychotic disorders. Eligible parturients were randomly assigned to the TAPB group (TAPB combined with PCIA with Nal and Dex) and the PCIA group (same block procedures with normal saline followed by PCIA with Nal and Dex) by using a computer‐generated random table. The study protocols were approved by the Ethics Committee of our hospital (No. K20240929 [EZ]) and performed in accordance with the Declaration of Helsinki. All participants provided written informed consent for study recruitment.

### 2.2 Spinal Procedures

When the parturient women arrived in the operating room, their intravenous accesses were opened, with their noninvasive blood pressure, peripheral oxygen saturation (SpO_2_), and electrocardiogram monitored. Spinal anesthesia was performed in the left recumbent position at the L3–4 interspace by using a 25‐G atraumatic needle. Following confirmation of clear cerebrospinal fluid flow, a dilution (3 mL) of 15 mg of 0.75% ropivacaine (AstraZeneca Pharmaceutical Co. Ltd., Beijing, China) added to 1 mL cerebrospinal fluid was injected. The operation was initiated after reaching T6 sensor blockade. All parturient women were managed by the same anesthesiologist, and none of them received any intraoperative opioids or sedatives.

### 2.3 Postoperative Analgesia Management

At the end of cesarean section, the parturient women in the TAPB group received ultrasound‐guided TAPB of 15 mg of 0.75% ropivacaine per side in the supine position in the anesthesia recovery room. Following skin disinfection, the ultrasound linear probe was perpendicularly placed on the mid‐axillary line between the iliac crest and subcostal margin. The transversus abdominis plane was identified between the oblique abdominis muscle and transversus abdominis muscle. A 22G sterile puncture needle (0.9 × 80 mm) was punctured into a vectorial plane about 3–4 cm medial to the ultrasound probe and then guided to the transversus abdominis plane. After confirmation of no blood pullback, 1 mL normal saline was injected to open the plane between the two muscles, followed by injection of 15 mL 0.75% ropivacaine. The other side of the transversus abdominis plane was blocked in the same way. The parturient women in the PCIA group underwent the same block procedures with normal saline. After the completion of surgery, the two groups of parturient women were connected to a patient‐controlled analgesia pump. The pump was programmed to intravenously administer 0.11 μg/kg/h Dex combined with 0.03 mg/kg/h Nal. The infusion rate was 4 mL/h, the bolus dose was 2 mL, and the lockout time was 15 min.

### 2.4 Outcomes

The primary outcome was postoperative pain degree evaluated by the visual analog scale (VAS) [15] at rest or mobilization at 2, 6, 12, 24, 36, and 48 h after surgery. The secondary outcomes were Ramsay sedation scale (RSS) scores [16] at 2, 6, 12, 24, 36, and 48 h after surgery, plasma cortisol (Cor) and norepinephrine (NE) levels, the first breast time, serum prolactin level, intraoperative SBP and DBP, the number of hypertension and hypotensive episodes, the time of analgesic demand on PCIA within 12 h after surgery, the first time of out‐of‐bed movement, the incidence of side effects within 24 h after anesthesia, such as nausea/vomiting, chills, dizziness, bradycardia/hypotension, urinary retention, skin pruritus, and respiratory depression defined as a respiratory rate < 10 or SpO_2_ < 90%, and maternal satisfaction. A four‐point scale was used to evaluate maternal satisfaction for postoperative pain control, with one point indicating dissatisfaction, two points indicating general satisfaction, three points indicating fair satisfaction, and four points indicating high satisfaction. The plasma level of Cor was determined by the radiation immune assay. The plasma level of NE was determined by the enzyme‐linked immunosorbent assay (ELISA) methods. The serum prolactin level was determined by the Elecsys Prolactin II assay (Roche Diagnostics International Ltd., Rotkreuz, Switzerland) using a Roche e602 autoanalyzer (Roche, Indianapolis, Indiana, USA).

### 2.5 Statistical Analysis

Sample size calculation was computed with prior power analysis using the G*power software (version 3.1.9.2) setting effect size as 0.25, *α* as 0.05, power as 0.9, and number of groups as 2, with loss of 10%. According to the normality of variable distribution confirmed by the Shapiro–Wilk test, the observed results were described as either mean ± standard deviation (SD) or median (quartile 1, quartile 3) and analyzed using the independent *t* test or Mann–Whitney *U* test between two groups. Moreover, categorical variables (*n*/%) were analyzed by the chi‐square test. All statistical tests used a two‐tailed *p* < 0.05 as statistically significant in GraphPad prism, version 6.0 (GraphPad, San Diego, CA, USA).

## 3 Results

### 3.1 Baseline Characteristics of Severely Pre‐Eclamptic Parturients

**FIGURE 1:**
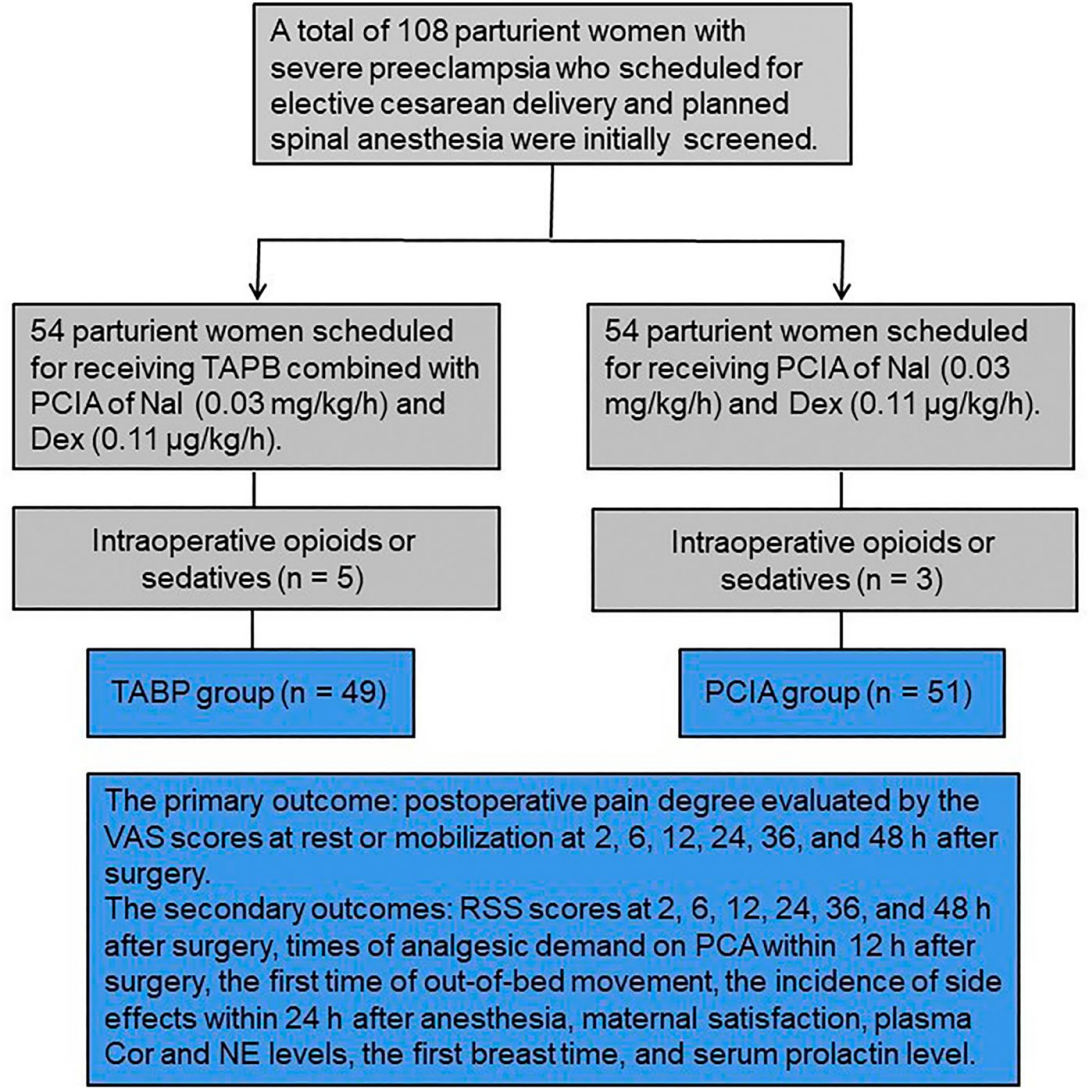
Flowchart of participant recruitment.

Fifty‐four severely pre‐eclamptic parturients who were scheduled for elective cesarean delivery and planned spinal anesthesia were initially enrolled for each group. According to the exclusion criteria, the study excluded 8 of the 108 enrolled for the use of intraoperative opioids or sedatives (Figure 1). The details of included parturient women are presented in Table 1 (*p* > 0.05).

**TABLE 1:** The details of included severely pre‐eclamptic parturients in the TAPB and PCIA groups.

	TAPB (*n* = 49)	PCIA (*n* = 51)	*p*
Maternal age	29.08 ± 4.14	30.04 ± 4.47	0.270
Maternal BMI (m^2^)	25.25 ± 2.87	25.71 ± 2.98	0.437
Gestational age (week)	38.61 ± 0.95	38.73 ± 1.10	0.564
Total operative time (min)	55.14 ± 15.68	54.16 ± 13.44	0.736
I‐D (h)	7.39 ± 4.59	8.51 ± 5.03	0.247
U‐D (h)	1.90 ± 0.94	2.08 ± 1.98	0.564
Intraoperative blood loss (mL)	353.10 ± 91.52	329.40 ± 96.53	0.212
Prepartum EPDS score	5 (4, 7)	5 (4, 6)	0.915
Birthweight (kg)	31.60 ± 0.14	31.65 ± 0.13	0.141
*Maternal education*			
Secondary school	2 (4.10%)	1 (2.00%)	0.630
High school	20 (40.82%)	25 (49.02%)	
University	27 (55.10%)	25 (49.02%)	
*Infant gender*			
Boy	27 (55.10%)	26 (50.98%)	0.680
Girl	22 (44.90%)	25 (49.02%)	

*Note:* Data summarized as mean ± SD are analyzed by the independent *t* test. Data summarized as median (quartile 1, quartile 3) are analyzed by Mann–Whitney *U* test. Data shown as numbers (percentage) are analyzed by the chi‐square test.

Abbreviations: I‐D, time interval from induction to delivery; PCIA, patient‐controlled intravenous analgesia; TAPB, transversus abdominis plane block; U‐D, time interval from uterus incision to delivery.

### 3.2 Postoperative Pain and Sedation Evaluation After Cesarean Section

**FIGURE 2:**
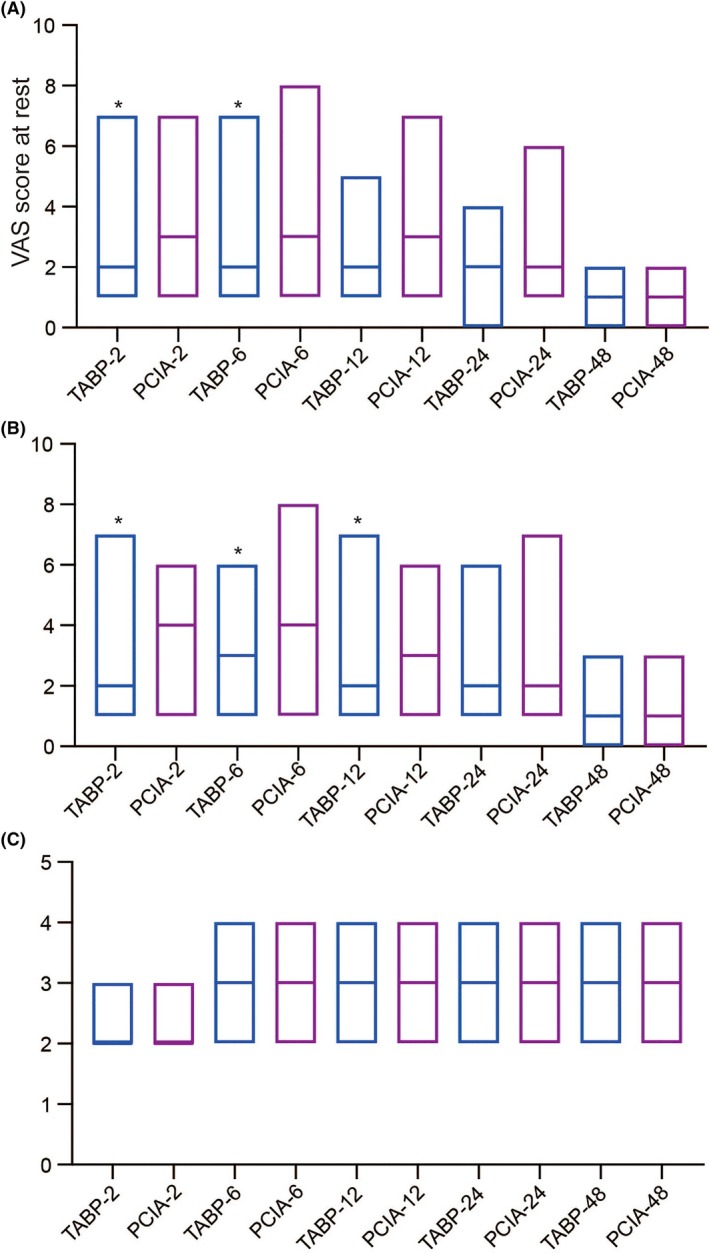
The VAS scores at rest and at mobilization and RSS scores recorded at 2, 6, 12, 24, and 48 h after surgery of severely pre‐eclamptic parturients in the TAPB and PCIA groups. Data summarized as median (quartile 1, quartile 3) are analyzed by Mann–Whitney *U* test. *p < 0.05 compared to the PCIA group.

The TAPB group exhibited lower VAS scores at rest recorded at 2 and 6 h following cesarean section than the PCIA group (*p* < 0.05, Figure 2A, Table 2). The TAPB group had decreased VAS scores at mobilization recorded at 2, 6, and 12 h following cesarean section than the PCIA group (*p* < 0.05, Figure 2B, Table 2). Two groups of parturients showed no significant difference in RSS scores at all four time points after surgery following cesarean section (*p* > 0.05, Figure 2C).

**TABLE 2:** The VAS scores at rest and at mobilization recorded at 2, 6, 12, 24, and 48 h after surgery of severely pre‐eclamptic parturients in the TAPB and PCIA groups.

	TAPB (*n* = 49)	PCIA (*n* = 51)	*p*
*VAS score at rest*			
T2	2 (1, 3)	3 (2, 4)	0.013
T6	2 (1, 4)	3 (2, 4)	0.024
T12	2 (1, 4)	3 (1, 4)	0.123
T24	2 (1, 3)	2 (1, 4)	0.255
T48	1 (0, 1)	1 (0, 1)	0.331
*VAS score at mobilization*			
T2	2 (1, 4)	4 (3, 5)	0.001
T6	3 (2, 4)	4 (3, 5)	0.003
T12	2 (1, 3)	3 (2, 4)	0.004
T24	2 (1, 3)	2 (1, 4)	0.156
T48	1 (1, 2)	1 (1, 2)	0.110

*Note:* Data summarized as median (quartile 1, quartile 3) are analyzed by Mann–Whitney *U* test.

Abbreviations: PCIA, patient‐controlled intravenous analgesia; TAPB, transversus abdominis plane block.

### 3.3 Stress Response and Lactation After Cesarean Section

**FIGURE 3:**
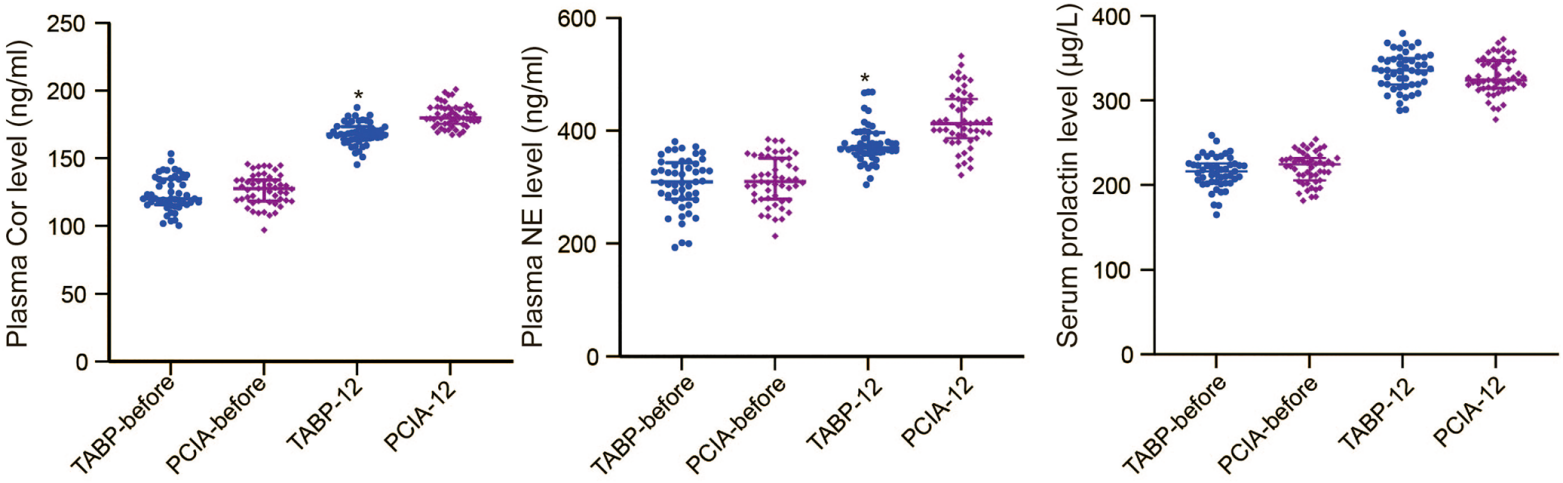
The plasma levels of Cor and NE and the serum level of prolactin before surgery and at 12 h after surgery of severely pre‐eclamptic parturients in the TAPB and PCIA groups. Data summarized as mean ± SD are analyzed by *t* test. *p < 0.05 compared to the PCIA group.

As shown in Figure 3, the plasma levels of Cor and NE were significantly increased at 12 h after cesarean section, but these increases were significantly greater in the PCIA group than in the TAPB group (*p* < 0.05). The first breast time in the TAPB group was earlier than that in the PCIA group (Table 3, *p* = 0.018). The serum level of prolactin did not differ between the TAPB and PCIA groups before surgery and at 12 h after surgery (Figure 3, *p* > 0.05).

**TABLE 3:** Maternal hemodynamic variables, drug consumption, and breast feeding after cesarean section between the TAPB and PCIA groups.

	TAPB (*n* = 49)	PCIA (*n* = 51)	*p*
The first breast time (h)	7 (5, 9.5)	9 (6, 11)	0.018
*Preoperative*			
SBP (mmHg)	155.8 ± 14.3	157.2 ± 13.7	0.624
DBP (mmHg)	100.4 ± 15.2	103.1 ± 13.6	0.372
*Intraoperative*			
Standardized SBP (mmHg)	125.4 ± 8.4	124.3 ± 6.4	0.476
Standardized DBP (mmHg)	80.3 ± 7.8	78.5 ± 6.7	0.208
Heart rate (beats/min)	83.5 ± 7.2	84.2 ± 6.9	0.638
Number of hypotensive episodes	1.5 (1–2)	2 (1–2)	0.493
*Postoperative*			
Number of hypotension	1	1	/
Press time of PCIA of Nal/Dex	1 (1, 2)	2 (1, 2)	0.014
Out‐of‐bed movement (h)	19.00 (16.80, 21.40)	20.20 (18.00, 22.50)	0.027

*Note:* Data summarized as mean ± SD or median (quartile 1, quartile 3) are analyzed by the independent *t* test or Mann–Whitney *U* test. Data shown as numbers (percentage) are analyzed by the chi‐square test.

Abbreviations: PCIA, patient‐controlled intravenous analgesia; TAPB, transversus abdominis plane block.

### 3.4 Maternal Hemodynamic Variables and Drug Consumption After Cesarean Section

The press times of demand on PCIA with Nal and Dex within 12 h after surgery, the first time of out‐of‐bed movement, and the incidence rate of side effects after surgery were compared between the TAPB and PCIA groups (Table 3). The TAPB group had fewer press times of demand on PCIA with Nal and Dex within 12 h after surgery than the PCIA group (*p* = 0.014). The first time of out‐of‐bed movement was notably shorter in the TAPB group than in the PCIA group (*p* = 0.027).

### 3.5 The Incidence of Side Effects and Maternal Satisfaction After Cesarean Section

The TAPB group had a lower incidence rate of side effects than the PCIA, but the difference was slight (12.24% vs. 23.53%, *p* > 0.05, Table 4). No adverse effects associated with TAPB were noted in the TAPB group. The total proportion of primiparous women with high satisfaction to pain control was notably higher in the TAPB group than in the PCIA group (*p* = 0.017, Table 4).

**TABLE 4:** The incidence of side effects and maternal satisfaction after cesarean section between the TAPB and PCIA groups.

	TAPB (*n* = 49)	PCIA (*n* = 51)	*p*
*Side effect*	6 (12.24%)	12 (23.53%)	0.194
Nausea/vomiting	2	4	
Chills	1	2	
Dizziness	1	2	
Bradycardia	1	1	
Urinary retention	0	1	
Skin pruritus	0	1	
Respiratory depression	1	1	
*Maternal satisfaction*			
Highly satisfied	29 (59.18%)	18 (35.29%)	0.017
Fairly satisfied	11 (22.45%)	15 (29.41%)	
General satisfied	6 (12.25%)	12 (23.54%)	
Dissatisfied	3 (6.12%)	6 (11.76%)	

*Note:* Data shown as numbers (percentage) are analyzed by the chi‐square test.

Abbreviations: PCIA, patient‐controlled intravenous analgesia; TAPB, transversus abdominis plane block.

## 4 Discussion

The main findings of our study were that adding TAPB significantly lowered VAS scores at 2 and 6 h after surgery, reduced press time of PCIA, shortened time for first feeding and out‐of‐bed movement, enhanced maternal satisfaction with pain control, and lowered plasma levels of Cor and NE.

Additional analgesic and opioid‐sparing options may be recommended for severely pre‐eclamptic parturients, those at risk of severe postoperative pain or in women failing to benefit complete postcesarean analgesia from standard analgesic regimes. TAPB has been demonstrated to reduce opioid consumption following cesarean section [17]. if TAPB is performed correctly for part of a multimodal analgesic scheme, parturients without pregnancy related disorders can possibly achieve effective pain control regarding somatic and visceral acute pain after cesarean section [18]. In our study, we demonstrated adding TAPB on PCIA of Nal/Dex could provide improved pain scores with movement within 12 h after surgery without adding side effects. Concurring with our results, Yan et al. [11] reported adding TAPB on intrathecal morphine could enhance early recovery after cesarean section in severely pre‐eclamptic parturients. Additionally, Joseph et al. [19] demonstrated use of TAPB could effectively both prolong analgesia and reduce the total consumption of analgesics for parturients without pregnancy related disorders after cesarean section. However, there have been controversial results of analgesic effects of TAPB. For example, Singh et al. [20] showed negative results that neither high‐dose nor low‐dose TAPB incorporating with intrathecal morphine improved postoperative pain at 24 h compared to placebo TAPB. Accordingly, in Kanazi et al.'s [21] study, they compared subarachnoid morphine with ultrasound‐guided TAPB after cesarean delivery and superior postcesarean analgesia, yet at the cost of additional incidence of side effects. Thus, TAPB incorporating with appropriate analgesic drugs to achieve complete postcesarean analgesia without adding side effects should be explored for a long time.

TAPB could alleviate the stress response after surgery [22]. In Qin et al.'s study [23], they found that the addition of 0.5 μg/kg Dex into ropivacaine for ultrasound‐guided TAPB could inhibit stress response with significantly declined serum levels of Cor and NE in patients undergoing laparoscopy gynecological surgery. The changes of Cor and NE are associated with perioperative stress. As shown in our study, adding TAPB on PCIA of Nal/Dex significantly lowered plasma levels of Cor and NE in severely pre‐eclamptic parturients after cesarean sections. A specific concern of TAPB in cesarean section is a toxicity risk due to the anesthetic passage to the neonate through maternal breast milk [24]. However, anesthetic drugs delivered in breast milk caused by TAPB is not high enough to lead to neonatal toxicity [25]. In this study, adding TAPB on PCIA of Nal/Dex significantly shortened time for first breast for severely pre‐eclamptic parturients after cesarean sections.

The application of TAPB could provide stable hemodynamics in the perioperative period [26]. Spinal anesthesia may contribute to an increased risk of hypotension compared to epidural anesthesia in severely pre‐eclamptic parturients, while the large prospective study still supports the use of spinal anesthesia for cesarean delivery in severe pre‐eclampsia [27]. According to a previous study, superior analgesia and lower incidence rates of hypotension and motor blockade were noted in TAPB as opposed to placebo block up to 48 h after surgery [28]. In this study, we did not observe intraoperative SBP and DBP, the numbers of intraoperative and postoperative hypertension and hypotensive episodes with or without TAPB, indicating additional TAPB did not cause maternal hemodynamics in severely pre‐eclamptic parturients with spinal anesthesia.

TAPB shows beneficial effects on opioid‐sparing perioperative management without increasing pain scores or decreasing patient satisfaction, which is an important component of enhanced recovery after surgery (ERAS) protocols for cesarean section [29, 30]. The use of TAPB in the setting of a well‐structured ERAS protocol was associated with a 36% reduction in narcotic use and a notable decline in length of stay by 1.5 days [31]. TAPB in conjunction with an opioid‐sparing analgesia in the ERAS program is feasible and effective in postoperative pain control during laparoscopic colorectal surgery [32]. Our data showed that adding TAPB to PCIA of Nal/Dex could enhance acute recovery for severely pre‐eclamptic parturients after cesarean sections, indicating the potential role of adding TAPB to PCIA with Nal and Dex in opioid‐sparing perioperative management for cesarean section in the context of preeclampsia.

We believed uncovering the study limitation and stating further prospective research could help to interpret our data. A relatively small sample size and subjects from a single center should be noted. We will expand the sample size in multicenters in the future to develop more choices of multimodal analgesia to achieve complete postcesarean analgesia. Further investigations by means of different approaches for TAPB or setting different doses of local anesthetics with adjuncts to prolong analgesic duration up to 48 h after surgery are warranted. More outcomes reflecting the interplay between mothers and newborns may aid in evaluating the safety of this multimodal analgesia protocol.

In conclusion, our study coincides well with the unique value of TAPB on severe pre‐eclampsia with respect to better postcesarean analgesia and enhanced acute recovery with less stress response. We confirmed that adding TAPB on PCIA with Nal and Dex could enhance acute recovery with less stress response for severely pre‐eclamptic parturients after cesarean delivery.

## Conflicts of Interest

The authors declare no conflicts of interest.

## Data Availability

Data sharing is not applicable to this article as no new data were created or analyzed in this study.
